# Defence transcriptome assembly and pathogenesis related gene family analysis in *Pinus tecunumanii* (low elevation)

**DOI:** 10.1186/s12864-018-5015-0

**Published:** 2018-08-23

**Authors:** Erik A. Visser, Jill L. Wegrzyn, Alexander A. Myburg, Sanushka Naidoo

**Affiliations:** 1 Department of Biochemistry, Genetics and Microbiology, Forestry and Agricultural Biotechnology Institute (FABI), Genomics Research Institute (GRI), University of Pretoria, Private bag X20, Pretoria, 0028 South Africa; 2 Department of Ecology and Evolutionary Biology, University of Connecticut, Storrs, CT 06269 USA

**Keywords:** *Pinus tecunumanii*, *Pinus patula*, Transcriptome assembly, PR genes, *Fusarium circinatum*, Disease resistance

## Abstract

**Background:**

*Fusarium circinatum* is a pressing threat to the cultivation of many economically important pine tree species. Efforts to develop effective disease management strategies can be aided by investigating the molecular mechanisms involved in the host-pathogen interaction between *F. circinatum* and pine species. *Pinus tecunumanii* and *Pinus patula* are two closely related tropical pine species that differ widely in their resistance to *F. circinatum* challenge, being resistant and susceptible respectively, providing the potential for a useful pathosystem to investigate the molecular responses underlying resistance to *F. circinatum*. However, no genomic resources are available for *P. tecunumanii*. Pathogenesis-related proteins are classes of proteins that play important roles in plant-microbe interactions, e.g. chitinases; proteins that break down the major structural component of fungal cell walls. Generating a reference sequence for *P. tecunumanii* and characterizing pathogenesis related gene families in these two pine species is an important step towards unravelling the pine-*F. circinatum* interaction.

**Results:**

Eight reference based and 12 de novo assembled transcriptomes were produced, for juvenile shoot tissue from both species. EvidentialGene pipeline redundancy reduction, expression filtering, protein clustering and taxonomic filtering produced a 50 Mb shoot transcriptome consisting of 28,621 contigs for *P. tecunumanii* and a 72 Mb shoot transcriptome consisting of 52,735 contigs for *P. patula*. Predicted protein sequences encoded by the assembled transcriptomes were clustered with reference proteomes from 92 other species to identify pathogenesis related gene families in *P. patula, P. tecunumanii* and other pine species.

**Conclusions:**

The *P. tecunumanii* transcriptome is the first gene catalogue for the species, representing an important resource for studying resistance to the pitch canker pathogen, *F. circinatum*. This study also constitutes, to our knowledge, the largest index of gymnosperm PR-genes to date.

**Electronic supplementary material:**

The online version of this article (10.1186/s12864-018-5015-0) contains supplementary material, which is available to authorized users.

## Background

The pitch canker fungus *Fusarium circinatum* Nirenberg and O’Donnell [1] has resulted in losses in pine plantations, seed orchards and nurseries worldwide [2, 3]. With a host range of more than 60 *Pinus* spp. [2], many of which are commercially important, this pathogen poses a significant threat to both forestry and conservation. The wide range of inter- and intraspecific variation in susceptibility of *Pinus* spp. to *F. circinatum* [4, 5] holds the potential for effective disease management through genetic resistance.

Development of more resistant families and genotypes for susceptible *Pinus* spp. [6] as well as selection and generation of resistant hybrids [7] have shown promise for long-term management of *F. circinatum*. Breeding and selection approaches, however, are time consuming and use of resistant genotypes could select for novel pathotypes [2]. Knowledge of the molecular mechanisms underlying resistance could expedite development of resistant genotypes and improve the effectiveness of genetic resistance.

The majority of information related to plant-pathogen interactions originate from studies on model plant species such as *Arabidopsis thaliana* and *Nicotiana* spp. [8]. Comparatively few studies have investigated these interactions in trees, and even less in gymnosperms. The main barrier to studying defence responses in non-model organisms in the past was the need for a reference genome. Among plants, particularly gymnosperms, genome size and complexity hindered sequencing and assembly of a reference genome. The availability of next-generation sequencing technology has circumvented this barrier for non-model organisms by enabling transcriptome assembly from high-throughput RNA sequencing (RNA-seq) data [9, 10].

While recent transcriptomic studies investigated the *Pinus*-*F. circinatum* interaction [11, 12], both studies focussed on susceptible species. Knowledge of defence mechanisms in the resistant interaction is lacking. The low elevation provenance (LE) of Tecun Uman Pine (*P. tecunumanii* Eguiluz & J. P. Perry) is an economically important *F. circinatum* resistant [4] *Pinus* species that has shown promise in hybridisation trials with *P. patula*, making it a good candidate for use as a model of resistance.

Studies on model plants have shown that molecular defence mechanisms consist of complex, multi-level processes [8]. In short, pathogen recognition occurs either through membrane binding of bound recognition receptors (PRRs) to pathogen−/damage−/microbe-associated molecular patterns (PAMPs, DAMPs, MAMPs) or through interaction between host resistance (R) genes and pathogen secreted effector proteins [13–15]. Pathogen perception results in activation of signal transduction cascades that initiate various local and systemic host defence responses including: reactive oxygen species (ROS) generation, cell wall modification, phytohormone defence pathways, defence-related protein expression and induction of systemic acquired resistance (SAR) [16–21]. An important group of markers for SAR are the pathogenesis-related (PR) proteins, a group of proteins identified due to their induction during biotic stress and direct antimicrobial activity of some PR gene family members [21–23]. There are currently 17 described PR gene families, classified by amino acid sequence and enzymatic activity, numbered in order of their description [22, 23]. Despite being the first family identified, a potential mode of action for the PR-1 proteins in plant defence was only recently described [24]. PR-1 proteins were shown to bind and sequester sterols, directly inhibiting sterol-auxotrophic pathogens as well as sterol-prototrophic pathogens with compromised sterol biosynthesis [24]. The PR-9 family are peroxidases that could be involved in cell wall reinforcement through catalysing lignification [25]. This could result in enhanced resistance against multiple pathogens [22, 23]. There are also two putative novel PR gene families, the PR-18 carbohydrate oxidases identified from *Helianthus annuus* and *Lactuca sativa* [26], and the PR-19 anti-microbial peptides identified from *Macadamia integrifolia* and *Pinus sylvestris* [27, 28].

This study aimed to produce a reference sequence for *P. tecunumanii* transcriptome profiling and a comparable reference for *P. patula*, as a resource for further investigation of the *Pinus*-*F. circinatum* host pathogen interaction, and to use the generated resources to identify the PR-gene families within these species. High quality reference transcriptomes were assembled, including the first gene catalogue for *P. tecunumanii* to date. These references were used to identify 639 and 785 PR gene candidates in *P. tecunumanii* and *P. patula* respectively.

## Methods

### Plant material and inoculation trial

Four month old low elevation (LE) *Pinus tecunumanii* seedlings, representing four open pollinated families, were sourced from SAPPI (Shaw Research Centre, South Africa) and 4 month old *P. patula* seedlings from a single open pollinated family were sourced from Mondi Forests (Trahar Technology Centre, South Africa). Seedlings were maintained in the Forestry and Agricultural Biotechnology Institute (FABI) *Fusarium* screening greenhouse at the University of Pretoria experimental unit. Pathogen challenge was performed as described in Visser et al. [12]. Briefly, **s**eedlings were inoculated with *F. circinatum* isolate FCC3579, harvested from cultures on ½ strength potato dextrose agar (½ PDA; Merck) washed with 15% (*v*/v) sterile glycerol and diluted to 5 × 10^4^ spores/mL, or mock-inoculated with 15% (v/v) sterile glycerol, at 6 months old by clipping the apical bud and depositing 10 μL inoculum on the wound. Tissue was harvested from seedlings at three and 7 days post inoculation (dpi) for three biological replicates (BR) per group. The top 1 cm of shoot tissue, from the point of inoculation, from 16 seedlings was pooled for each biological replicate. Harvested tissue was flash frozen in liquid nitrogen and stored at − 80 °C until use. Disease progression was monitored by measuring stem length and stem discolouration from the point of inoculation over 6 weeks to calculate the percentage live stem ([stem length (mm)-stem discolouration (mm)]/stem length (mm)). The difference in mean percentage live stem between inoculated and mock-inoculated plants at each time point was analysed using a Kruskal-Wallis rank sum test (*p* < 0.05). Re-isolation of the pathogen was performed by placing tissue harvested at 14 dpi on ½ PDA and observing culture morphology after 7 days.

### RNA isolation and sequencing

Total RNA was extracted from homogenised samples, ground in a mortar and pestle, using the Plant/Fungi RNA Purification Kit (Norgen Biotek Corp., Thorold, ON, Canada) according to the manufacturer’s instructions, with the inclusion of acid washed glass beads during lysis to improve cell break down. Extracted samples were assessed using a Bio-Rad Experion™ automated electrophoresis system (Bio-Rad Laboratories, Hercules, CA, USA) to determine sample concentration as well as ensure sample integrity (RNA Integrity Number > 7.0) and purity (absence of genomic DNA).

Inoculated and mock-inoculated samples, for both time points, were sent to Novogene (Novogene Corporation Inc., Chula Vista, CA, USA) for strand specific RNA-Sequencing on an Illumina HiSeq2500 (Illumina, San Diego, CA, USA). Samples were sequenced in three sets for each species, to optimise read length for transcriptome assembly (Additional file 1: Table S1). Sample set one consisted of a single sample of pooled RNA from all six three dpi samples (500 bp insert, PE250 sequencing). Sample set two consisted of the six samples (3 BR inoculated, 3 BR mock-inoculated) for three dpi (300 bp insert, PE125 sequencing). Sample set three consisted of the six samples (3 BR inoculated, 3 BR mock-inoculated) for seven dpi (300 bp insert, PE150 sequencing).

### Transcriptome assembly and annotation

RNA-sequencing libraries obtained from Novogene were assessed using FastQC [29]. Preliminary transcriptome assembly was performed using Trinity v2.2.0 [30], on two datasets (Additional file 1: Table S1). Reads were quality trimmed and filtered using trimmomatic v0.32 (Additional file 1: Table S2) [31]. For each species, all 13 trimmed and filtered libraries were combined to produce a full dataset. In silico read normalisation, to a maximum coverage of 100, was performed using Trinity on the full dataset to produce a normalised dataset. Both datasets were used for transcriptome assembly. Twenty strand specific preliminary assemblies were produced, with a minimum contig length of 350, using two different: *k*-mer sizes (25 & 31), stringencies for de Bruijn graph construction, and algorithms for transcript reconstruction (Additional file 1: Table S2). The normalised dataset for both species was mapped against the *P. taeda* v1.01 draft genome assembly using GSNAP 2016-11-07 (Genomic Short Read Alignment Program; Additional file 1: Table S2) [32] for genome guided assembly. Preliminary assemblies were filtered to obtain the longest isoform per locus, an assembly code (Additional file 1: Table S2) added to the transcript identifiers, and combined to form a highly redundant assembly. Redundancy was reduced using the EvidentialGene [33] tr2aacds pipeline v2016.07.11. Assembly statistics were calculated using Transrate v1.0.3 [34].

Primary transcripts from the tr2aacds pipeline were annotated using the eukaryotic non-model transcriptome annotation pipeline v0.7.3.2 (EnTAP) [35]. In short, the normalised dataset was mapped to the transcripts using Bowtie2 v2.3.0 [36]. Transcript expression was calculated using RSEM v1.3.0 (RNA-Seq by Expectation-Maximum) [37] and transcripts with an FPKM < 1 (Fragments Per Kilobase of transcript per Million mapped reads) were discarded. Frame prediction was performed on transcripts with detectable expression using GeneMarkS-T v5.1 March 2014 [38], transcripts without a predicted reading frame were discarded, and predicted proteins clustered to 90% identity using Usearch v9.0.2132 [39]. Diamond v0.8.31 [40] was used to obtain protein BLAST alignments for predicted protein sequences of the remaining transcripts against: the RefSeq Complete Protein database, the UniProt-KB/Swissprot database, and the *Arabidopsis thaliana* proteome; using a minimum query coverage of 80%, a minimum target coverage of 60% and a minimum e-value of 1e-4. Only the best hits across all three databases were retained. Functional annotation, Gene Ontology (GO) annotation relative to the full GO database and orthologous group assignment, was performed using EggNOG 4.5 [41]. Unannotated sequences were discarded as erroneously assembled transcripts. Non-pine origin sequences were removed based on best hit taxonomy (discarding sequences with best hits from archaea, fungi, insects, bacteria, viruses and vertebrates), for sequences with BLASTp alignments, and orthologous group taxonomic scope (discarding sequences not associated with Viridiplantae, Eukaryota or Ancestor lineages), for sequences without BLASTp alignments, to produce the *P. tecunumanii* (Pnte_v1.0) and *P. patula* (Pipt_v2.0) draft transcriptome assemblies. For ease of comparison, GO terms were normalised to level two of the classification tree. Putative TAIR10 identifiers were assigned to transcripts based on best hits to the *A. thaliana* proteome. Completeness and contiguity of the assembly was determined using BUSCO v3.0 (Benchmarking of Single-Copy Orthologs; [42]) against the eukaryote_odb9 (303 BUSCOs) and embryophyta_odb9 (1440 BUSCOs) lineages and compared to 26 other gymnosperm assemblies (Additional file 1: Table S3) obtained from the TreeGenes database [43].

### Orthogroup identification

Orthologous gene clusters (orthogroups) were identified using OrthoFinder v1.1.10 [44] across 94 different species (Additional file 1: Table S4), using default settings, similar to previous studies [45, 46]. The dataset consisted of proteomes for: the red algae *Cyanidioschyzon merolae* as outgroup, 11 species of green algae, the liverwort *Marchantia polymorpha*, the moss *Physcomitrella patens*, the clubmoss *Selaginella moellendorffii*, 14 gymnosperm species (including Pnte_v1.0 and Pipt_v2.0), the basal angiosperm *Amborella trichopoda*, 18 monocot species and 46 dicot species, containing a total of 2,974,043 protein sequences. The proteomes for *P. taeda*, *P. lambertiana* and *Pseudotsuga menziessii* were obtained from the TreeGenes database [43]. All remaining proteomes were retrieved from the PLAZA database. Orthogroups containing previously identified putative PR-genes for *Arabidopsis thaliana*, *Brachypodium distachyon*, *Oryza sativa*, *Populus trichocarpa* and *Vitis vinifera* [47] were classified as pathogenesis-related gene families for PR-1 through PR-17. Putative PR-18 and PR-19 orthogroups were identified through BLASTp alignment of type sequences to the OrthoFinder input dataset. Both *H. annuus* and *L. sativa* carbohydrate oxidase protein sequences (Genbank accessions AAL77103.1 and AAL77102.1) [26] were used as type sequences for identification of putative PR-18 orthogroups. Putative PR-19 orthogroups were identified using four *P. sylvestris* antimicrobial peptide sequences (Genbank accessions AAL05052.1 to AAL05055.1) as well as the first antimicrobial peptide identified from *M. integrifolia* (UniProtKB/Swiss-Prot accession P80915.1).

## Results and discussion

### *F. circinatum* disease progression on *P. tecunumanii* and *P. patula*

**Fig. 1:**
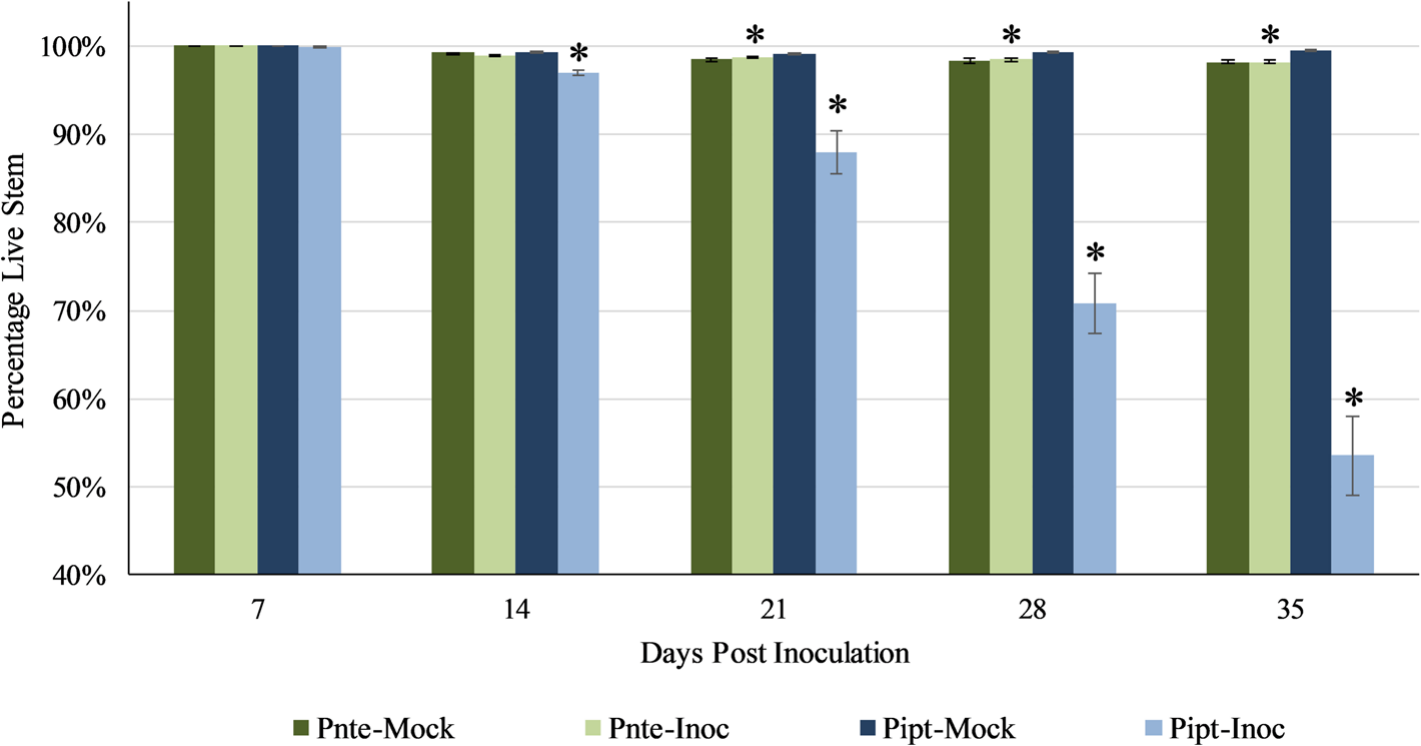
*Fusarium circinatum* disease progression on inoculated low elevation *Pinus tecunumanii* and *Pinus patula* seedlings. Error bars represent the standard error of the mean (Inoc *n* = 100; Mock *n* = 20). Pnte – *P. tecunumanii*; Pipt – *P. patula*; Inoc – inoculated; Mock – Mock-inoculated; * - significant difference between inoculated and mock-inoculated groups (Kruskal-Wallis rank sum test, *p* < 0.05)

Seedlings of *P. tecunmanii* and *P. patula* were inoculated with *F. circinatum* and the decline in percentage green stem monitored over the course of 6 weeks (Fig. 1). Stem discolouration, at the point of inoculation, was visible on all seedlings at 7 days post inoculation with no discernible difference in lesion colour or length between inoculated and mock-inoculated seedlings. By 14 dpi a clear difference in lesion colouration was visible between treatment groups for both species, with inoculated seedlings displaying purple lesions, and a significant difference in lesion length was observed between *P. patula* treatment groups (*p* < 0.05). Initial mortality of *P. patula* seedlings was observed at 21 dpi. By 42 dpi chlorosis prevented further measurements of *P. patula* seedlings. While a significant difference in percentage green stem was observed for *P. tecunumanii* seedlings after 14 dpi, this was due to more wound discolouration on mock-inoculated relative to inoculated seedlings and all seedling had more than 98% live stem. The difference in stem discolouration between *P. tecunumanii* and *P. patula* is therefore consistent with the classification of *P. tecunumanii* as a resistant host [4].

### Transcriptome assembly

Illumina sequencing and subsequent filtering by Novogene produced ca. 530- and ca. 570 million clean read pairs for *P. tecunumanii* (LE) and *P. patula*, respectively (Additional file 1: Table S1). Following read trimming and filtering, the *P. tecunumanii* (LE) full dataset contained a total of ca. 870 million reads (81.6% of the clean reads) representing ca. 120 Gb of sequence; the *P. patula* full dataset contained ca. 950 million reads (83.5% of clean reads) representing ca. 130 Gb of sequence. Normalisation to 100X read coverage retained ca. 54 million reads (6.2% of the full dataset) in the normalised dataset for *P. tecunumanii* (LE) and ca. 77 million reads (8.1% of the full dataset) in the normalised dataset for *P. patula*.

**Fig. 2:**
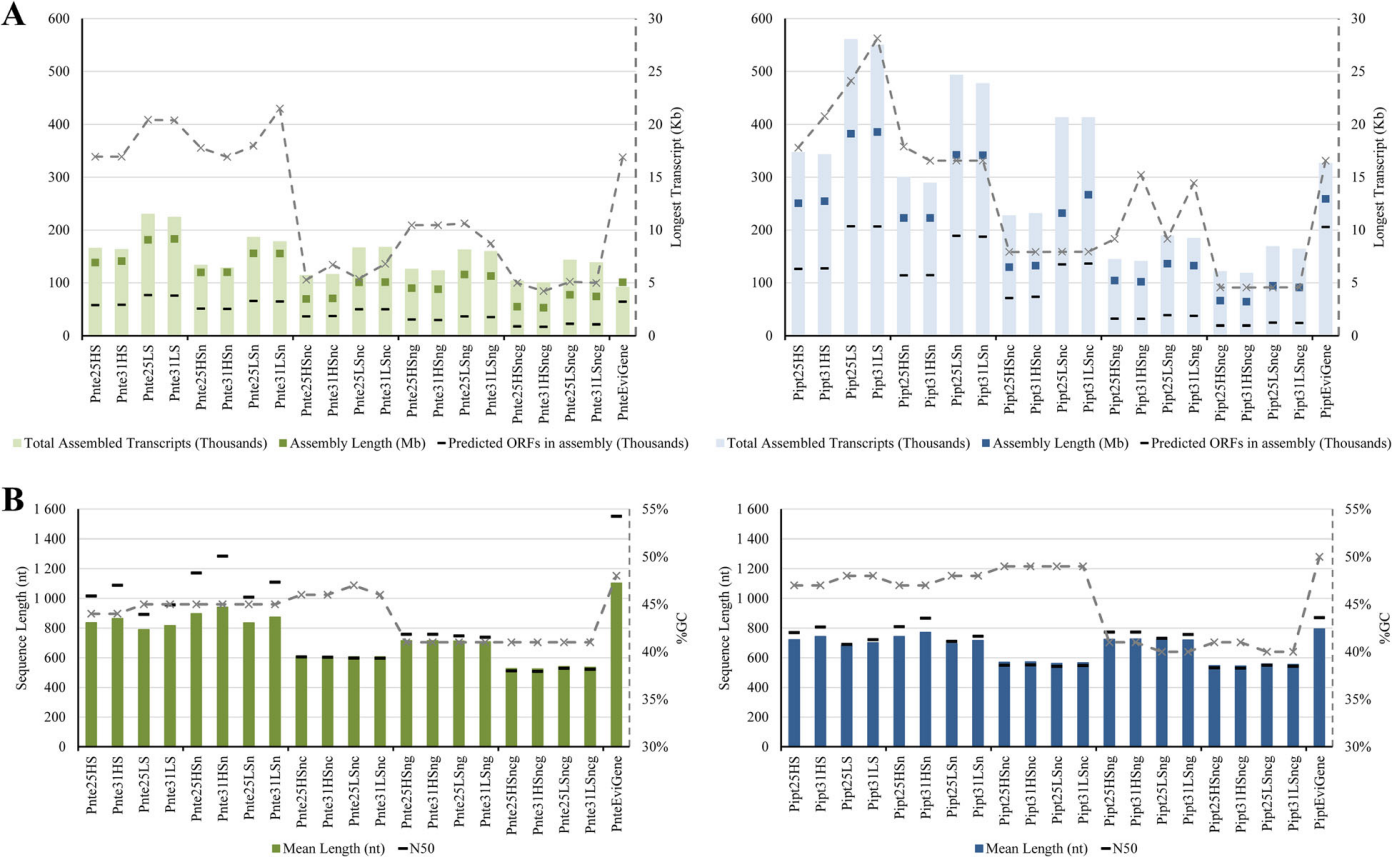
Summarised statistics for the longest isoforms per gene for preliminary assemblies. Left – *Pinus tecunumanii* (LE) assemblies (Pnte). Right – *Pinus patula* assemblies (Pipt). **a** Size, length, predicted open reading frame (ORF) content and longest transcript per assembly statistics. **b** N50, mean length and %GC. The grey, dashed, secondary y-axes only applies to the dashed grey lines. The x-axes represent respective assemblies. In each case the first four assemblies were produced using the full dataset and the remaining 16 with the normalised dataset. Numbers in assembly identities (25 & 31) represent the *k*-mer size used, ‘c’ indicates usage of CuffFly transcript reconstruction algorithm, ‘g’ indicates genome guided assemblies, ‘n’ indicates usage of the normalised dataset, ‘HS’ and ‘LS’ indicate usage of high- and low-stringency parameters respectively

Four de novo assemblies, with two different *k*-mer lengths and stringencies, were constructed using the full dataset (Fig. 2). The normalised dataset was used to construct eight de novo and eight genome guided assemblies; using two different *k*-mer lengths, stringencies and reconstruction algorithms. Preliminary assembly generated 20 transcriptomes containing a total of 3,023,703 transcripts for *P. tecunumanii* (LE) and 5,868,982 transcripts for *P. patula*, after filtering assemblies to only retain the longest isoform per gene (Fig. 2).

Redundancy across the preliminary transcripts was reduced based on coding potential using the tr2aacds pipeline from EvidentialGene. Following open reading frame (ORF) prediction, 36.2% and 37.4% of input sequences were classified as non-redundant, for *P. tecunumanii* (LE) and *P. patula* respectively, of which 29.6% and 37.4% were retained as informative after substring dereplication using CD-HIT-EST v4.6.1 [48, 49]. Removal of low quality transcripts resulted in 182,681 sequences grouped into 91,552 loci for *P. tecunumanii* (LE), defined as the PnteEviGene assembly, and 542,459 sequences grouped into 325,974 loci for *P. patula*, defined as the PiptEviGene assembly (Fig. 2). When compared to the *P. tecunmanii* (LE) Trinity assemblies the PnteEviGene assembly showed a better ratio of assembled transcripts to overall assembly length as reflected by its high N50 and mean contig length, which was greater than any of the input assemblies, indicating that the EvidentialGene pipeline successfully selected the longest assembled isoforms from among the Trinity assemblies. For *P. patula* on the other hand, comparison of the EviGene assembly to the Trinity assemblies showed less of an improvement. Still, the PiptEviGene assembly, similar to the PnteEviGene assembly, had a higher average transcript length and N50 than any of the Trinity assemblies.

### Annotation

Expression based filtering of the PnteEviGene and PiptEviGene assemblies retained ca. 85% and ca. 63% of transcripts (Table 1). GeneMarkS-T successfully predicted coding regions for ca. 95% of expressed transcripts for both species, though the *P. patula* assembly contained a ca. 10% lower proportion of complete reading frames compared to the *P. tecunumanii* assembly. Protein clustering retained ca. 77% of the EvidentialGene transcripts for *P. tecunumanii*, while only ca. 51% of *P. patula* transcripts were retained, indicating that greater redundancy was retained in the *P. patula* assembly during CD-hit-EST clustering.

**Table 1:** Summarised EnTAP annotation statistics

	*Pinus tecunumanii* (LE)		*Pinus patula*	
Assembly Statistics				
Assembly	PnteEviGene	Pnte_v1.0	PiptEviGene	Pipt_v2.0
Total Sequences	91,552	28,621	325,974	52,735
Total Transcriptome Length (Mb)	100.95	50.01	259.02	72.15
Average Sequence Length (nt)	1103	1747	794	1368
N50 (nt)	1551	2296	870	1897
Longest Sequence (nt)	16,886	16,886	16,570	16,570
Shortest Sequence (nt)	351	351	351	351
%GC	48%	44%	50%	46%
Sequence filtering^a^				
Sequences with FPKM > 1	77,563		203,996	
	*(84.72%)*		*(62.58%)*	
Sequences with GeneMarkS-T predicted CDS	74,556		194,568	
	*(81.44%)*		*(59.69%)*	
Total proteins after clustering to 90% identity	70,748		167,961	
	*(77.28%)*		*(51.53%)*	
Annotation^b^				
	Predicted protein frame			
Complete	32,193	18,971	61,134	26,835
	*(45.50%)*	*(66.27%)*	*(36.40%)*	*(50.89%)*
Internal	18,279	3347	57,027	9602
	*(25.84%)*	*(11.70%)*	*(33.95%)*	*(18.21%)*
3′-partial	6686	1895	21,848	4354
	*(9.45%)*	*(6.63%)*	*(13.01%)*	*(8.26%)*
5′-partial	17,398	4408	54,559	11,944
	*(24.59%)*	*(15.40%)*	*(32.48%)*	*(22.65%)*
	Similarity Search Annotation			
Sequences with informative BLASTp alignments	19,296	15,192	38,714	27,328
	*(27.27%)*	*(53.09%)*	*(23.05%)*	*(51.82%)*
Sequences with uninformative BLASTp alignments	15,437	6131	34,101	9261
	*(21.82%)*	*(21.41%)*	*(20.30%)*	*(17.56%)*
Sequences without BLASTp alignments	36,015	7300	95,146	16,147
	*(50.91%)*	*(25.50%)*	*(56.65%)*	*(30.62%)*
	Functional Annotation			
Sequences with family assignment	55,627	28,484	128,952	52,166
	*(78.63%)*	*(99.52%)*	*(76.77%)*	*(98.92%)*
Sequences with at least one GO term	31,640	16,197	77,063	33,712
	*(44.72%)*	*(56.60%)*	*(45.88%)*	*(63.93%)*
Sequences with at least one pathway (KEGG) assignment	17,303	8004	47,383	21,094
	*(24.46%)*	*(27.98%)*	*(28.21%)*	*(40.00%)*
	Annotation Summary			
Unannotated Sequences	14,568	0	36,698	0
	*(20.59%)*	*(0.00%)*	*(21.85%)*	*(0.00%)*
Total sequences annotated	56,180	28,621	131,263	52,735
	*(79.41%)*	*(100.00%)*	*(78.15%)*	*(100.00%)*
Non-pine origin sequences	27,550	0	78,527	0
	*(38.94%)*	*(0.00%)*	*(46.75%)*	*(0.00%)*

^a^Percentages relative to EviGene assemblies

^b^Percentages relative to clustered GeneMarkS-T assemblies for EviGene columns and relative to total sequences for Pnte_v1.0 and Pipt_v2.0

Best hit selection of BLAST alignments, *P. tecunumanii* (*P. patula*) clustered protein sequences, to *Arabidopsis*, RefSeq and UniProt resulted in informative hits for ca. *27*% (23%) of contigs, uninformative hits for ca. 22% (20%) of contigs and no hits for ca. 51% (57%) of contigs. EggNOG functional annotation assigned ca. 79% (77%) of contigs to orthologous groups. In total, ca. 79% (78%) of contigs were successfully annotated. Taxonomy based filtering identified 27,550 (78,527) non-pine origin contigs.

Removal of non-pine origin contigs produced the first *P. tecunumanii* draft transcriptome (Pnte_v1.0), containing 28,621 contigs of which ca. 53% had informative BLAST annotations and ca. 99% were assigned to EggNOG functional annotations (Additional file 2: Table S6). The current *P. patula* draft transcriptome (Pipt_v2.0) contained 52,735 contigs of which ca. 52% and 99% had informative BLAST annotations and EggNOG functional annotations respectively (Additional file 3: Table S7), an improvement on the 60% annotation of Pipt_v1.0 [12]. TAIR identifiers could be assigned to 16,393 (ca. 57%) Pnte_v2.0 and 27,954 (ca. 53%) Pipt_v2.0 contigs (Additional file 4: Table S8, Additional file 5: Table S9). Best hit annotation of non-pine origin sequences indicated that the majority of contaminants were of fungal origin (Additional file 1: Table S5).

**Fig. 3:**
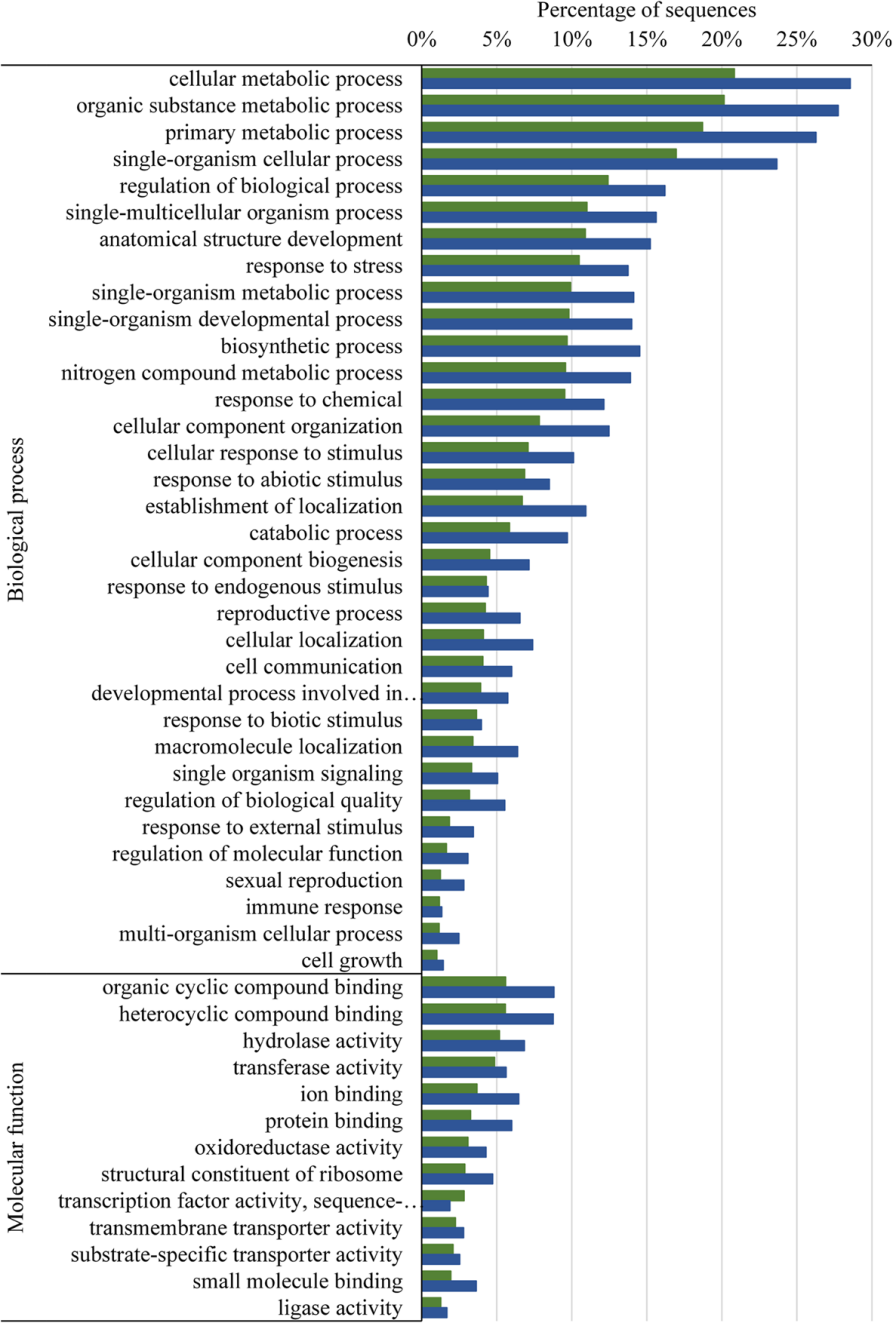
Distribution of biological process and molecular function Gene Ontology (GO) terms in the assembled transcriptomes. Green – *Pinus tecunumanii* assembly. Blue – *Pinus patula* assembly

Gene Ontology (GO) terms were assigned to ca. 57% (16,197) of *P. tecunumanii* contigs; 11,157 contigs with biological process (BP) terms, 8086 contigs with molecular function (MF) terms, and 15,077 contigs with cellular compartment (CC) terms (Table 1). For *P. patula*, GO terms were assigned to ca. 64% (33,712) of contigs; 24,709 contigs with BP terms, 18,956 contigs with MF terms and 31,760 contigs with CC terms. Among the top MF terms identified for both species were hydrolase activity, transferase activity, ion binding, protein binding and two parent terms for nucleic acid binding (organic cyclic acid binding, heterocyclic compound binding), similar to what has been observed for other conifer reference sequences [11, 12, 50, 51] (Fig. 3). The most common BP terms were indicative of rapid and extensive metabolic activity within the analysed tissue, as has been shown for the interaction between *P. radiata* and *F. circinatum* [11].

**Fig. 4:**
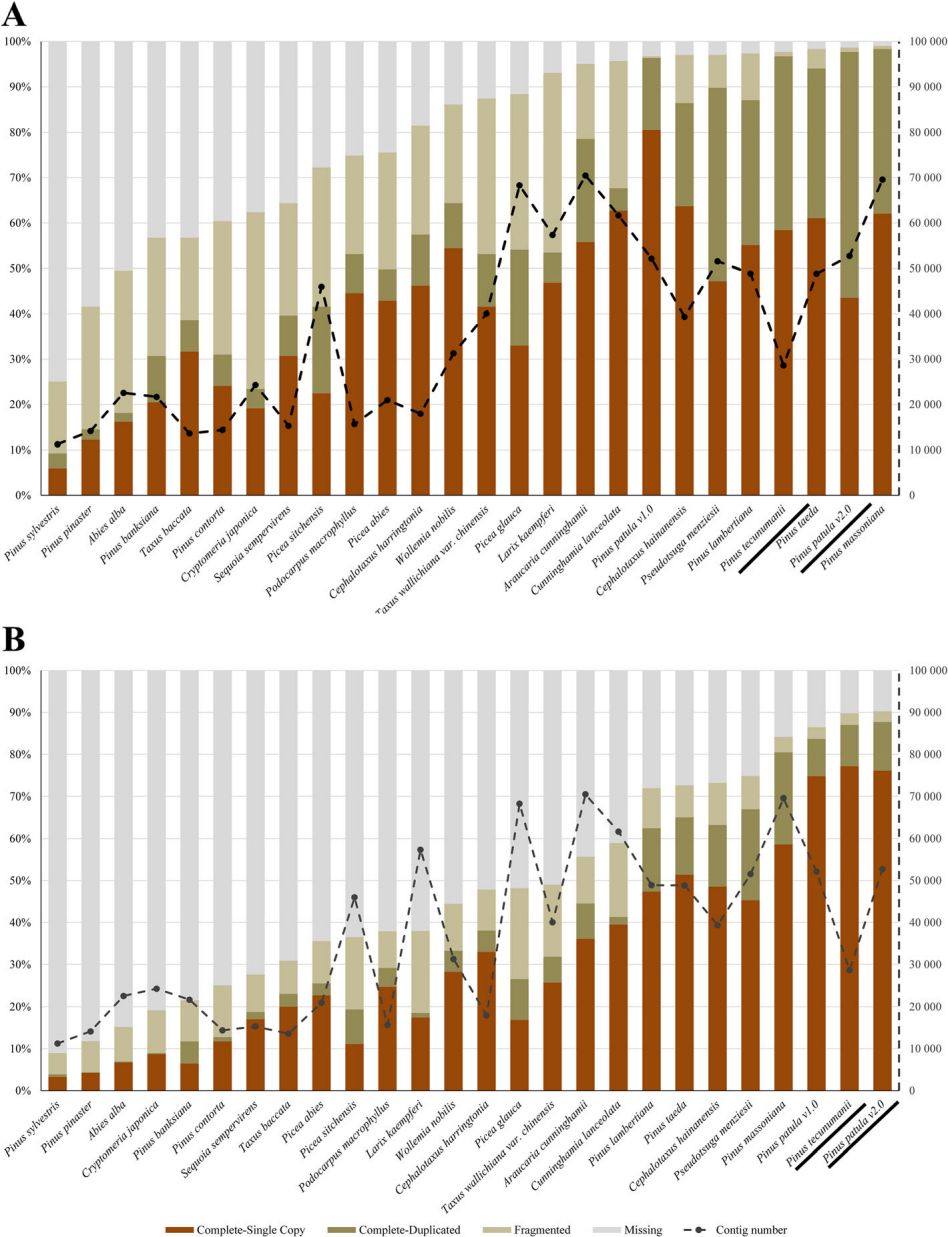
Comparison of completeness, contiguity and redundancy for assembled transcriptomes (underlined) to other available gymnosperm assemblies. BUSCOs were identified for the (**a**) eukaryotic (*n* = 303) and (**b**) embryophyta (*n* = 1440) lineage datasets. The primary y-axis refers to the percentage of BUSCOs in each category. The secondary, dashed, y-axis refers to the total contig count in each assembly

Completeness (total complete and fragmented BUSCOs), contiguity (total complete BUSCOs/total complete and fragmented BUSCOs) and redundancy (duplicated BUSCOs) of the assemblies was determined by comparison to the BUSCO eukaryote (*n* = 303) and embryophyte (*n* = 1440) lineage datasets [42] (Fig. 4). When compared to the eukaryotic lineage the BUSCO results for Pnte_v1.0 and Pipt_v2.0 (C:96.7%,[S:58.4%,D:38.3%],F:1.0%,M:2.3% and C:97.7%,[S:43.6%,D:54.1%],F:1.0%,M:1.3%; where C refers to the percentage of complete BUSCOs, S refers to the percentage of complete and single copy BUSCOs, D refers to complete and duplicated BUSCOs, F refers to fragmented BUSCOs and M refers to missing BUSCOs) showed high completeness (97.7% and 98.7%) and contiguity (99.0% and 99.0%) for both assemblies. While redundancy (38.3% and 54.1%) was also high for both assemblies, a similar trend was observed for the other gymnosperm assemblies analysed (Fig. 4). Comparison to the embryophyte lineage (Pnte_v1.0 = C:87.0%,[S:77.2%,D:9.9%],F:2.8%,M:10.2% and Pipt_v2.0 = C:87.7%,[S:76.1%,D:11.6%],F:2.6%,M:9.7%) showed lower redundancy (9.9% and 11.6%). The completeness (89.8% and 90.3%) and contiguity (96.9% and 97.2%) of Pnte_v1.0 and Pipt_v2.0 for the embryophyte lineage was the highest amongst analysed assemblies. The high redundancy in the assemblies likely reflects assembled haplotypes [42] due to the high genetic variance present in the populations from which the data was generated, indicating that more variance was present in the *P. patula* seedlings compared to the *P. tecunumanii* (LE) seedlings.

### Identification of pathogenesis-related gene families

A total of 51,594 orthogroups were identified, containing 2,599,567 genes, of which 248 groups contained genes from all 94 species. For Pnte_v1.0, 28,298 (98.8%) contigs were assigned to 9561 orthogroups, of which 4, containing 8 contigs, were species specific. For Pipt_v2.0, 50,244 (95.3%) contigs were assigned to 11,325 orthogroups, of which 85, containing 225 contigs, were species specific. Of the total orthogroups, 9071 were Gymnosperm specific. A further 7072 were specific to conifers, of which 3181 were specific to pines (Additional file 6: Figure S1).

PR genes identified in *A. thaliana*, *B. distachyon*, *P. trichocarpa*, *O. sativa* and *V. vinifera* [47] were used to identify putative PR orthogroups for 16 of the 17 currently classified plant PR classes, as well as the two putative novel classes (Additional file 1: Table S10). PR-15 and PR-16 were both classified as PR-16 due to their high homology and the classification of PR-15 as monocot specific [52]. Putative PR orthogroups were identified for 15 PR families in *P. tecunumanii* and 16 PR families in *P. patula* (Additional file 1: Table S11). PR-12 and -13 members were absent from both assemblies. Both of these appear to be angiosperm specific as the PR-12 orthogroup only contained sequences from the basal angiosperm *A. trichopoda* and dicots, while the PR-13 orthogroup only contained sequences from monocots and brassicaceae, similar to what has previously been observed [47, 53]. The PR-6 family was absent from the *P. tecunumanii* assembly, indicative of insufficient expression for assembly rather than absence from the genome. Two putative PR-6 genes were identified in the *P. patula* assembly, interrogation of EggNOG annotations identified the “potato-inhibitor I family domain” found in the PR-6 type member in both sequences.

The only PR classes for which putative members were present in all species were PR-2 and PR-9, although no single orthogroup was present in all species. Putative PR-1 orthologues were only absent from some of the chlorophyte species. PR-7 members were present for all viridiplantae species with PR-8, − 11 and − 18, while not identified for all species, similarly present across all viridiplantae lineages. The PR-10 and PR-17 orthogroups only contained sequences from embryophyte species and the PR-14 orthogroup only contained tracheophyte sequences. While some chlorophyte sequences were present, the PR-3, − 5, and − 16 orthogroup sequences were mostly spread evenly between the embryophyte lineages. The majority of identified PR-4 and -6 sequences were identified in angiosperms. Despite being initially identified from a dicot, putative PR-19 sequences, as expected, were only identified in the lycophyte *S. moellendorfii*, coniferous gymnosperms, the basal angiosperm *A. trichopoda* and in low numbers in monocots [54].

**Fig. 5:**
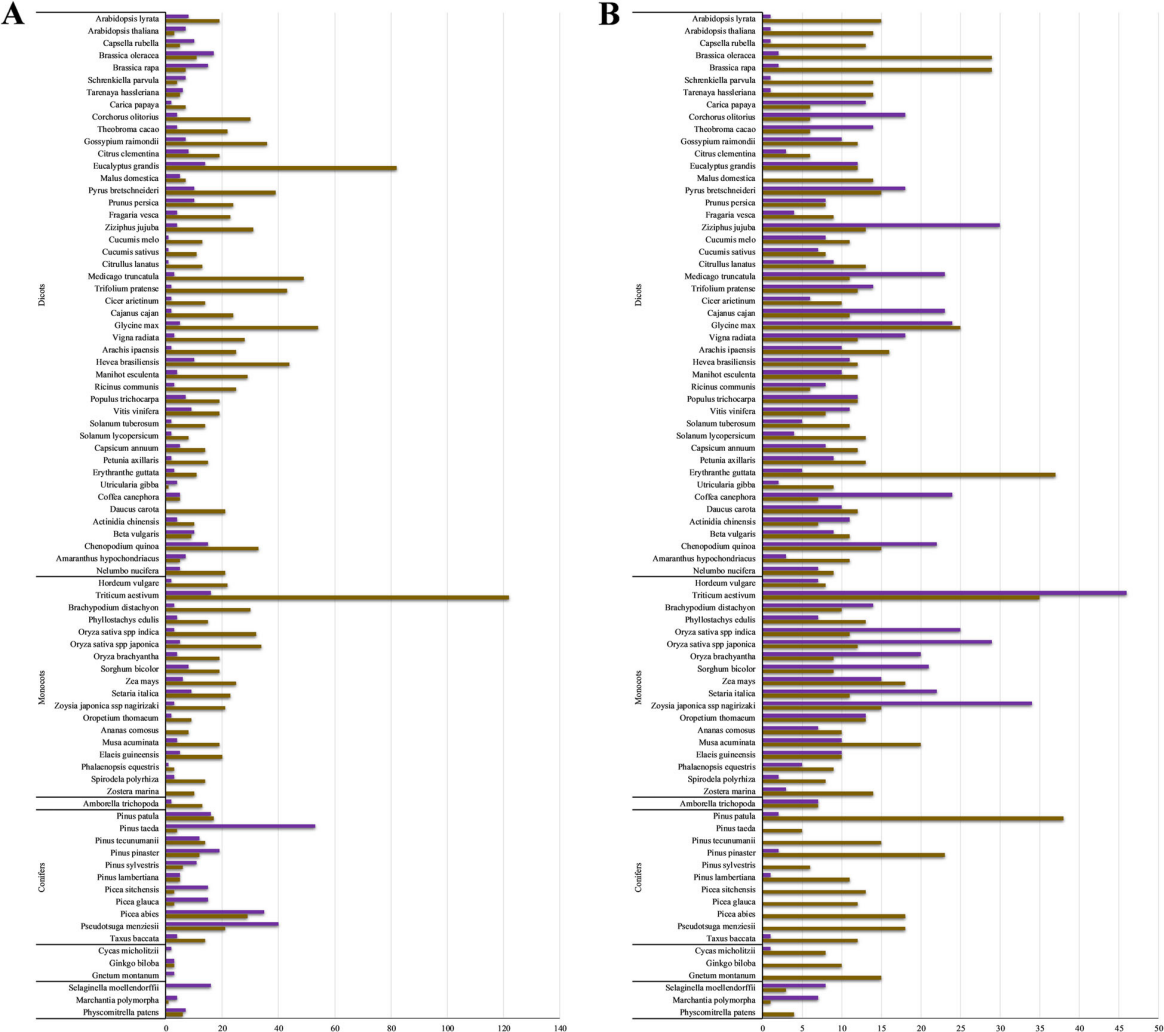
Comparison of orthogroup size across lineages between two chitinase orthogroups. **a** PR-3 orthogroups OG0000134 (brown) and OG000642 (purple). **b** PR-8 orthogroups OG0000252 (brown) and OG0000455 (purple). In both cases the x-axis refers to the amount of proteins per species present in each orthogroup

Potential lineage specific chitinase gene family expansions were observed when comparing two of the identified PR-3 orthogroups (OG0000134 and OG0000642), as well as the two PR-8 orthogroups identified (Fig. 5). When looking at the PR-3 orthogroups, both groups appear present in relatively equal amounts in gymnosperms, while OG0000134 is more prominent in angiosperms. The opposite is seen for the two PR-8 groups, with both relatively similar in angiosperms but OG0000252 more prominent in gymnosperms. Interestingly, in both cases the pattern observed for the brassicales is similar to the gymnosperms not the angiosperms.

**Fig. 6:**
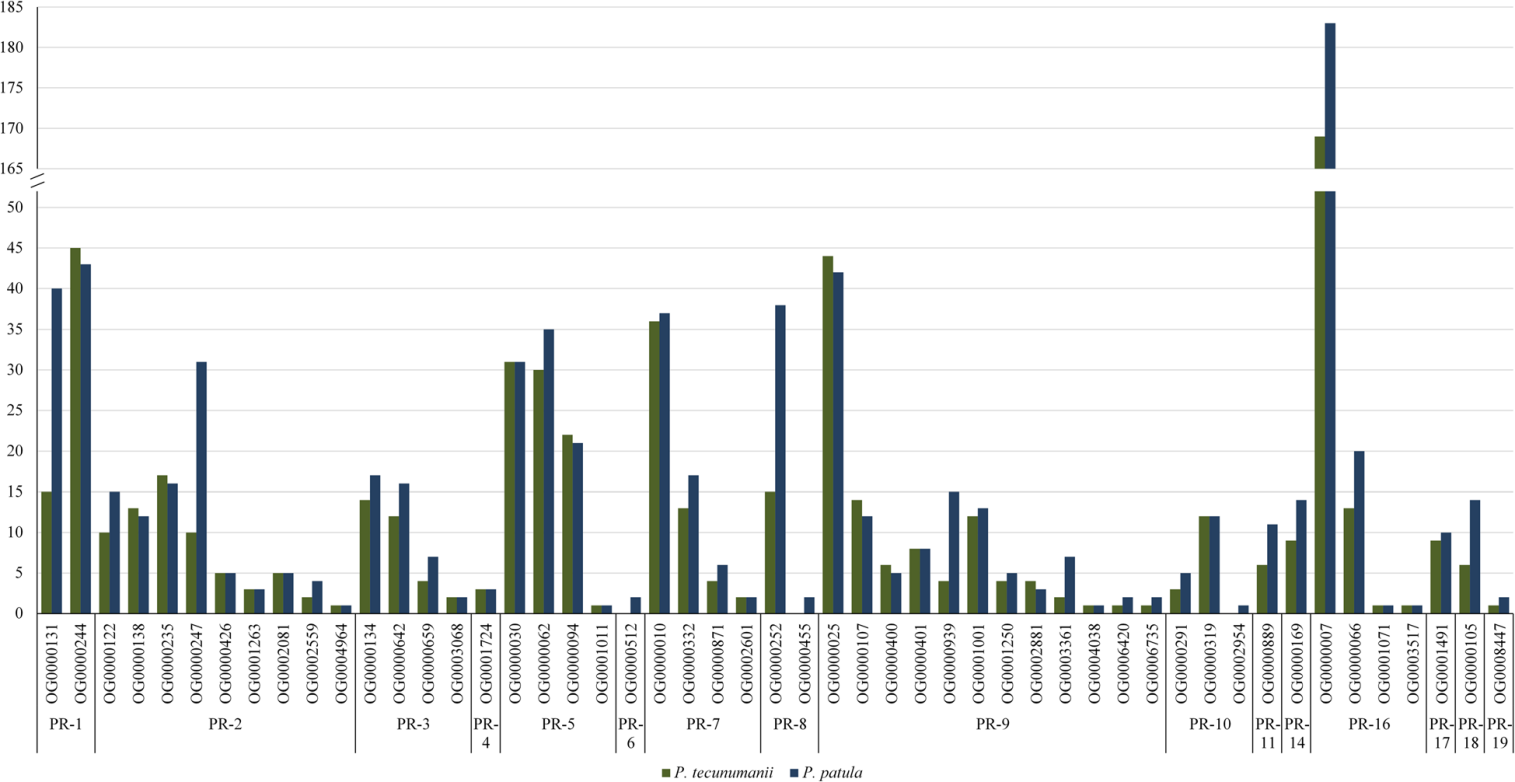
Putative Pathogenesis Related orthogroup transcript counts in *P. tecunumanii* and *P. patula*. The y-axis represents the transcript count for each orthogroup

More putative PR genes were identified in *P. patula* (801) relative to *P. tecunumanii* (646). While lower amounts of *P. tecunumanii* transcripts in an orthogroup is likely due to the difference in the number of transcripts assembled, the reverse could indicate transcripts absent from the *P. patula* defence response. *P. patula* had more transcripts for all PR gene classes identified except the PR-4 chitinase family (OG0001724), for which 3 transcripts were identified in both species (Fig. 6). The number of transcripts identified for the PR-5, PR-10, PR-11, PR-14, PR-17, PR-18 and PR-19 families, were very similar between species, with at most five transcripts more in *P. patula*. When comparing the four PR-5 orthogroups present in both species, OG0000039 and OG0001011 had the same amount of transcripts and OG0000062 had more *P. patula* transcripts, while OG0000094 had one more *P. tecunumanii* transcript. Of the 51 PR orthogroups containing transcripts from either species, 3 only had *P. patula* transcripts, 26 had more *P. patula* than *P. tecunumanii* transcripts, 14 had the same amount of transcripts and only 8 had more *P. tecunumanii* transcripts. Similar to the PR-5 orthogroup (OG0000094), two PR-2 (OG0000138; OG0000235) and two PR-9 (OG0000400; OG0002881) orthogroups had one more *P. tecunumanii* transcript. An additional two PR-9 (OG0000025; OG0000107) and one PR-1 (OG0000244) orthogroup had two more transcripts from *P. tecunumanii* compared to *P. patula*. The relative expansion observed in four of the PR-9 peroxidase orthogroups could indicate a more robust cell wall reinforcement or oxidative burst response in *P. tecunumanii*, however, this remains to be functionally determined*.*

## Conclusions

In summary, we report the assembled juvenile shoot transcriptome for *Pinus tecunumanii*, the first reference sequence for this species, as well as a comparable juvenile shoot transcriptome for *P. patula*. Both assemblies represent important resources that will contribute to further study of the *Pinus*-*F. circinatum* interaction. Furthermore, of the 19 PR classes, putative homologues for 15 were identified in *P. tecunumanii* and for 16 in *P. patula*, resulting in a total of 646 and 801 putative PR genes respectively. This work provides a critical base for future investigation of host-pathogen interactions in these tropical pine species as well as characterisation of other, non-defence related molecular pathways. The assembled transcriptomes will be used as reference to investigate host expression during *F. circinatum* challenge, allowing comparison of resistant and susceptible host responses between closely related species. In addition, the transcriptomes could be used to help characterise genetic markers for these tropical pines.

## Additional files

Additional file 1: **Table S1.** RNA-sequencing library statistics. **Table S2.** Program parameters used for assembly that deviated from the default. **Table S3.** BUSCO input transcriptome sources. **Table S4.** OrthoFinder input proteome sources. **Table S5.** Best hit species distributions. **Table S10.** Putative PR gene family counts. **Table S11.** Putative *Pinus tecunumanii* and *Pinus patula* PR genes. (XLSX 110 kb)

Additional file 2: **Table S6.** Pnte_v1.0 annotation. (TSV 48464 kb)

Additional file 3: **Table S7.** Pipt_v2.0 annotation. (TSV 124607 kb)

Additional file 4: **Table S8.** Pnte_v1.0 TAIR identifiers. (TSV 2028 kb)

Additional file 5: **Table S9.** Pipt_v2.0 TAIR identifiers. (TSV 3421 kb)

Additional file 6: **Figure S1.** Orthologous group distribution across analysed lineages. Secondary and tertiary Venn diagrams were constructed based on the lineage specific orthogroups. (TIFF 2590 kb)

## Abbreviations

BLAST: Basic Local Alignment Search Tool
BP: Biological process
BR: Biological replicate
BUSCO: Benchmarking Universal Single Copy Orthologs
CC: Cellular compartment
CDS: Coding DNA sequence
DAMP: Damage Associated Molecular Pattern
DEG: Differentially Expressed Gene
Dpi: Days post inoculation
EnTAP: eukaryotic non-model Transcriptome Annotation Pipeline
FPKM: Fragments Per Kilobase of transcript per Million mapped reads
Gb: Gigabase
GO: Gene Ontology
GSNAP: Genomic Short-read Nucleotide Alignment Program
KEGG: Kyoto Encyclopedia of Genes and Genomes
LE: Low elevation
MAMP: Microbe Associated Molecular Pattern
Mb: Megabase
MF: Molecular function
ORF: Open reading frame
PAMP: Pathogen Associated Molecular Pattern
PDA: Potato dextrose agar
PR genes: Pathogenesis-Related genes
PRR: Pattern Recognition Receptor
R gene: Resistance gene
RNA-seq: RNA sequencing
ROS: Reactive oxygen species
RSEM: RNA Sequencing by Expectation Maximisation
SAR: Systemic acquired resistance
TAIR: The Arabidopsis Information Resource
