# Supplementary figures and images for: Defence transcriptome assembly and pathogenesis related gene family analysis in Pinus tecunumanii (low elevation)
Source: BMC Genomics. 2018 Aug 23;19:632. doi: 10.1186/s12864-018-5015-0 (PMC6108113; doi:10.1186/s12864-018-5015-0)

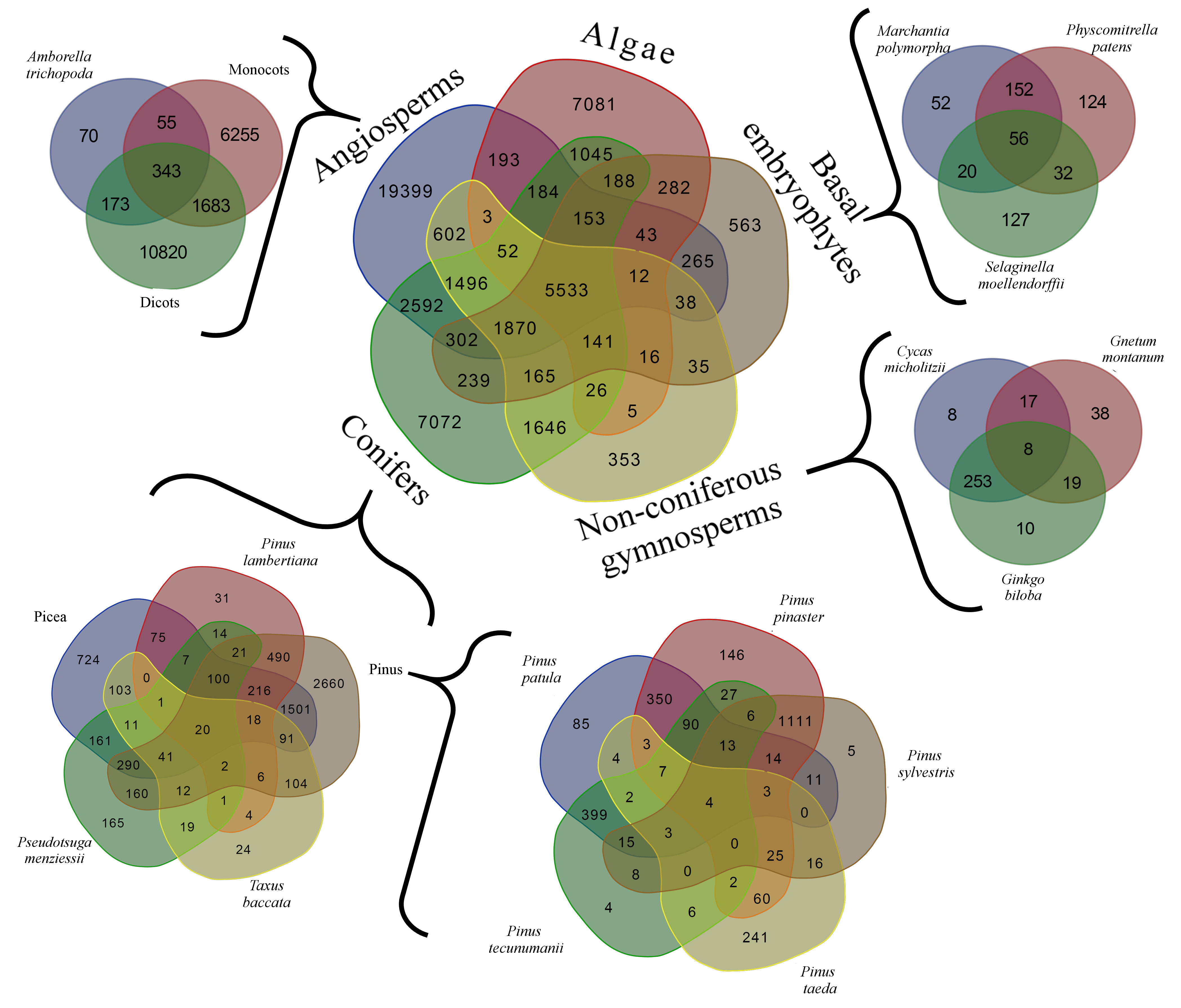

Supplement: Supplementary file 6 — Figure S1. Orthologous group distribution across analysed lineages. Secondary and tertiary Venn diagrams were constructed based on the lineage specific orthogroups. (TIFF 2590 kb) [file 12864_2018_5015_MOESM6_ESM.tiff]
